# Path2enet: generation of human pathway-derived networks in an expression specific context

**DOI:** 10.1186/s12864-016-3066-7

**Published:** 2016-10-25

**Authors:** Conrad Droste, Javier De Las Rivas

**Affiliations:** Bioinformatics and Functional Genomics Group, Cancer Research Center (CiC-IBMCC, CSIC/USAL/IBSAL), Consejo Superior de Investigaciones Cientificas (CSIC), Salamanca, Spain

**Keywords:** Biological pathway, Protein network, Gene network, Network analysis, Transcriptomics, Expression, Gene coexpression, Bioinformatics, R package

## Abstract

**Background:**

Biological pathways are subsets of the complex biomolecular wiring that occur in living cells. They are usually rationalized and depicted in cartoon maps or charts to show them in a friendly visible way. Despite these efforts to present biological pathways, the current progress of bioinformatics indicates that translation of pathways in networks can be a very useful approach to achieve a computer-based view of the complex processes and interactions that occurr in a living system.

**Results:**

We have developed a bioinformatic tool called *Path2enet* that provides a translation of biological pathways in protein networks integrating several layers of information about the biomolecular nodes in a multiplex view. *Path2enet* is an R package that reads the relations and links between proteins stored in a comprehensive database of biological pathways, KEGG (Kyoto Encyclopedia of Genes and Genomes, http://www.genome.jp/kegg/), and integrates them with expression data from various resources and with data on protein-protein physical interactions. *Path2enet* tool uses the expression data to determine if a given protein in a network (i.e., a node) is active (ON) or inactive (OFF) in a specific cellular context or sample type. In this way, *Path2enet* reduces the complexity of the networks and reveals the proteins that are active (expressed) under specific conditions. As a proof of concept, this work presents a practical “case of use” generating the pathway-expression-networks corresponding to the NOTCH Signaling Pathway in human B- and T-lymphocytes. This case is produced by the analysis and integration in *Path2enet* of an experimental dataset of genome-wide expression microarrays produced with these cell types (i.e., B cells and T cells).

**Conclusions:**

*Path2enet* is an open source and open access tool that allows the construction of pathway-expression-networks, reading and integrating the information from biological pathways, protein interactions and gene expression cell specific data. The development of this type of tools aims to provide a more integrative and global view of the links and associations that exist between the proteins working in specific cellular systems.

**Electronic supplementary material:**

The online version of this article (doi:10.1186/s12864-016-3066-7) contains supplementary material, which is available to authorized users.

## Background

Large-scale “omic” experiments that capture the physical associations and links between genes, proteins and other molecular components within the cells are producing extensive data on biomolecular interactions which are stored in new generation databases and resources [1]. The human interactome, for example, is composed of around 20,000 protein-coding genes, around 1000 metabolites and a still undefined number of distinct proteins and functional RNA molecules [2]. In total, this sums up to more than 100,000 cellular components expected to form the complex machinery of human cells. These components are related to each other in different ways. The number of relations and functional associations substantially exceeds the number of components, making the interactome a large relational system difficult to depict and analyze. Despite this complexity, the nature of the cellular interactomes allows to render or transcribe them into biomolecular “networks” that can integrate different layers of information to generate comprehensive spaces, providing a better view of the cellular systems. Moreover, the “networks” can be analyzed with computers to explore and quantify the centrality and the weight of the different components, and to find clusters or modules of highly related elements. This is the framework that drived us to develop the bioinformatic application tool here presented, called *Path2enet*.

*Path2enet* is an R package that reads the relations and links between proteins stored in the major and highly curated pathways database KEGG (Kyoto Encyclopedia of Genes and Genomes) [3, 4] and integrates them with gene expression data from various resources as well as experimentally determined data from protein-protein physical interactions taken from APID [5, 6]. *Path2enet* tool uses the expression data to determine if a given node (protein) in a generated network is active (ON) or inactive (OFF) in a specific cellular context, cell type or condition. The transformation of pathways into comprehensive networks plus the mapping of active –i.e., expressed– nodes, can help researchers to integrate different levels of molecular information, placing it in a relational specific context. In addition, the integration of protein-protein interaction data within a pathway-network view can help to find relevant relations and critical nodes in the processes studied. As a practical example, we applied *Path2enet* tool to the analysis of the NOTCH Signaling Pathway in human lymphocytes in order to uncover the specific differences between B cells (CD19+) and T cells (CD4+ or CD8+).

## Methods

### Integration of pathways, molecular interactions and expression resources

*Path2enet* is an R package that uses and integrates several databases and resources to generate pathway-derived networks in an expression specific context. These resources are the following: (**A**) pathways data, the tool collects the pathways information from KEGG, taking the KGML-files and generating a MySQL database from such files (this data integration provides a set that contains 50,448 unique interactions for human) [3, 4]; (**B**) protein-protein interaction data, the tool also uses a dataset of human protein-protein physical interactions (PPIs) from the dataserver APID [6], which at the time of building the package contained 284,263 unique interactions of human proteins; and (**C**) gene expression data, the tool integrates four types of expression information. These are: (**C1**) ESTs (expressed sequence tags) from the Unigene database that includes 18,880 gene/protein entries detected in 51 human tissues (http://www.ncbi.nlm.nih.gov/unigene); (**C2**) *Barcode* gene expression from high-density oligonucleotide microarrays that store 17,268 gene/protein entries detected in 195 tissues and cell lines [7, 8]; (**C3**) RNA-Seq data of the Human Body Map 2.0 (ArrayExpress Experiment E-MTAB-513) that stores FPKM expression data of 18,744 gene/protein entries in 16 human tissues (these FPKMs –fragments per kilobase of exon per million reads– were calculated using *Cufflings 2.2.0* algorithm [9] and annotated to *Ensembl GRCh37* with the R-package *Biomart* [10]; and (**C4**) RNA-Seq data from the Human Protein Atlas which stores the FPKM expression data of 19,078 gene/protein entries of 33 human tissues (http://www.proteinatlas.org) [11].

### Calculation of expression level to identify ON/OFF genes

Beside the pre-processed expression datasets provided in several of the integrated resources, *Path2enet* uses the gene expression *Barcode* algorithm with the R package *fRMA* [8] to evaluate if a gene is expressed (i.e., is ON, active and present) or not (i.e., such gene is OFF, not-active and therefore not expressed) in a studied set of samples. The user can also incorporate and apply in *Path2enet* his own expression ON/OFF thresholds, for example using experimental RNA-Seq data. However, the identification of such thresholds is not trivial and the *Barcode* algorithm is most efficient in this task.

### ID mapping and data unification

For the ID mapping and integration, *Path2enet* uses *Brainarray* [12] or *Gatexplorer* [13] within R to annotate the probe-set identifiers of the microarrays to *Ensembl* gene identifiers.

To achieve a correct unification of databases and resources, *Path2enet* uses as key identifiers (IDs) of the genes/proteins the entry IDs from *UniProtKB* database [14]. Therefore, the KEGG gene and *Ensembl* gene identifiers in the datasets are annotated to the *UniProt* entry IDs using the mapping tables that *UniProt* provides. *Path2enet* also uses the R package *RMySQL* [15] to build and to connect to the *MySQL* databases using R programming. Finally, in order to build the networks, *Path2enet* uses the R package *igraph* [16], which is a tool that provides outputs that can be introduced in Cytoscape.

### Selection of an experimental dataset to apply *Path2enet*

As a practical example, we applied *Path2enet* to analyze the NOTCH Signaling Pathway in human lymphocytes, detecting the way in which this pathway is expressed in these cells and also finding the specific differences in activated genes/proteins between “naive” B cells (B cells that have not been exposed to an antigen) and T cells. To perform this analysis we downloaded and normalized an expression dataset that included 163 human samples. These samples were genome-wide expression microarrays of platform Human Genome U133 Plus 2.0 from *Affymetrix* (GEO reference: GPL570). The samples corresponded to naive B cells (CD19+), 32 microarrays; T cells (CD4+), 96 microarrays; and T cells (CD8+), 35 microarrays. The specific. CEL files (i.e., the raw data) that correspond to these samples are indicated in Additional file 1, and are available in the Gene Expression Omnibus (GEO) database from NCBI.

### Software availability and implementation

*Path2enet* has been developed in R (free software environment for statistical computing and graphics, https://www.r-project.org/). In this way, a full operative R package has been built and it is available at http://bioinfow.dep.usal.es/path2enet. The software will be uploaded to the R CRAN package repository (CRAN.R-project.org) once this article is published. An R vignette (enclosed as Additional file 2) is provided as a guided tutorial to facilitate the installation and use of the *Path2enet* package.

## Results and discussion

### Building networks and performing analysis with *Path2enet*

*Path2enet* is a bioinformatic application tool that integrates the information of pathways, protein-protein interactions and expression datasets (obtained with microarrays, RNA-Seq or ESTs) from different tissues and cell types. *Path2enet* uses these datasets to build a network view of biological pathways in an expression-specific context. The tool is capable of identifying the genes/proteins that are ON in specific samples appliying the *Barcode* algorithm, and allows the use of specific experimental expression data to present focused views of the human pathways map as specific biomolecular networks.

In the networks built using *Path2enet*, the “nodes” correspond to the proteins included in the queried pathway plus the information about the active- or inactive-state of such proteins (derived from the expression data of the cell-types or the tissues studied in each case). The “edges” of the network correspond to the links or associations between the biomolecular entities (derived from the information included in the pathways). These links can be activation, inhibition, expression, phosphorylation, etc. In order to facilitate further analysis of the networks, the edges generated by *Path2enet* are taken as undirected.

**Fig. 1:**
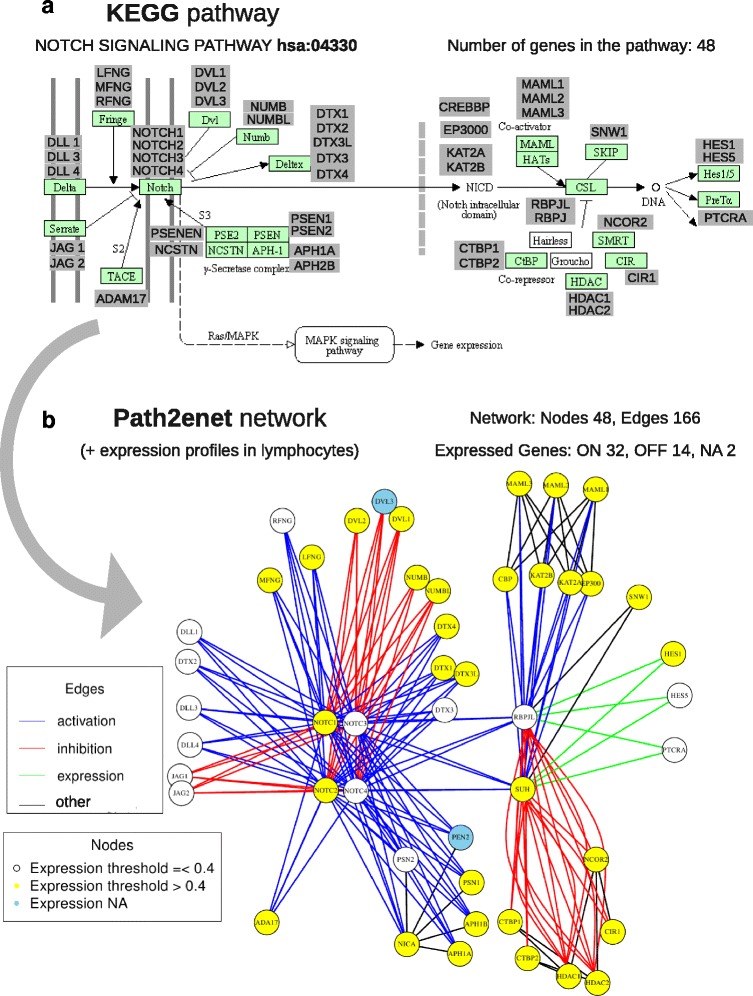
Figure showing the NOTCH Signaling Pathway and the transformation into a network using *Path2enet*: (**a**) the canonical map included in the KEGG database (hsa:04330), showing all the distinct proteins that are included in each node of this pathway (total 48 proteins); (**b**) transfromation of the canonical pathway to a network done with *Path2enet*, that produces a new view incorporating all the information about the gene products that are expressed (i.e., active) based on the pathway and on the expression data sets of lymphocytes that are incorporated (163 samples: 32 B cells and 131 T cells). *Path2enet* uses the gene expression *Barcode* algorithm to evaluate if a gene is expressed (ON, yellow) in the network or not (OFF, white). The genes in blue correspond to NA (not assigned) since the tool could not assign them because they are not present in the expression platform used. The panels inside the figure indicate: "Edges" the characteristics of the links/relations that connect each node (blue, activation; red, inhibition; green, expression; black, other type of link); "Nodes" the expression level assigned to each node in the sample type studied that in this case were lymphocytes (white nodes, when their expression is below the threshold ≤0.4; yellow nodes, when their expression is above the threshold >0.4; blue nodes, when the expression level is not assigned)

Considering the coverage over the map of human pathways, *Path2enet* can generate two different types of networks. The first is the “local” network which strictly includes the nodes of the canonical pathway selected from KEGG. For example, in the case of the NOTCH Signaling Pathway (KEGG ID: hsa04330) (Fig. 1a) the “local” network retrieves the 48 genes/proteins that are included in this pathway for human (*Homo sapiens*). Thus, *Path2enet* generates a network where each node is a protein and the edges are colored according to the type of association reported in the pathway (Fig. 1b). The second type of network that *Path2enet* can build is the “global” pathway-network that includes all the “local” nodes and links from a given KEGG pathway, plus all the extra “external” nodes that such nodes can be linked to in other pathway charts (i.e., it provides the links to other nodes in any biological pathway of the whole human repertoire). In this way, *Path2enet* is not restricted to predefined pathways since it can create large networks blending multiple layers of biological information.

Once a network is built with *Path2enet*, calculations of the network topological parameters (such as degree, betweeness, clustering coefficient, eigenvector value, etc.) can be performed, because the tool generates *igraph* objects [16], that can be studied with graph analysis tools. In this way, *Path2enet* provides ways to identify hubs and clusters in the network.

### Application of *Path2enet* to build the NOTCH pathway-network of B and T cells

**Fig. 2:**
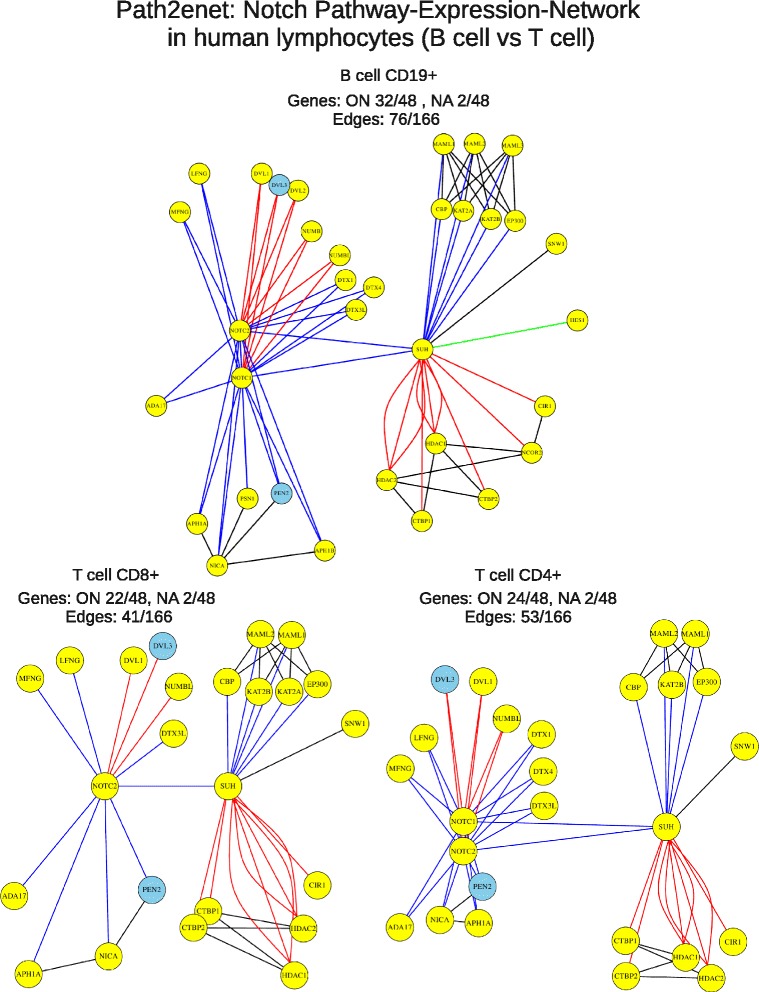
Pathway-expression-networks produced for B cell CD19+ (top), T cell CD8+ (bottom left) and T cell CD4+ (bottom right) based on the transformation of the NOTCH Signaling Pathway done with *Path2enet*. The tool removed all nodes below the expression treshhold of 0.4. The expression network produced for B cells reveals 32 active nodes (ON) plus 2 NAs out of 48 proteins and 76 edges (out of 166 maximum). The expression network for T cells reveals 22–24 active nodes (ON) plus 2 NAs out of 48 proteins and 41–53 edges (out of 166 maximum)

In the case study presented in this article we used *Path2enet* to generate expression networks of the NOTCH signaling pathway in three types of human cells: B cells (CD 19+) and T cells (CD 4+ and CD8+) (Fig. 2). To achieve this, we used a sample dataset of microarray expression (indicated in Methods).

**Fig. 3:**
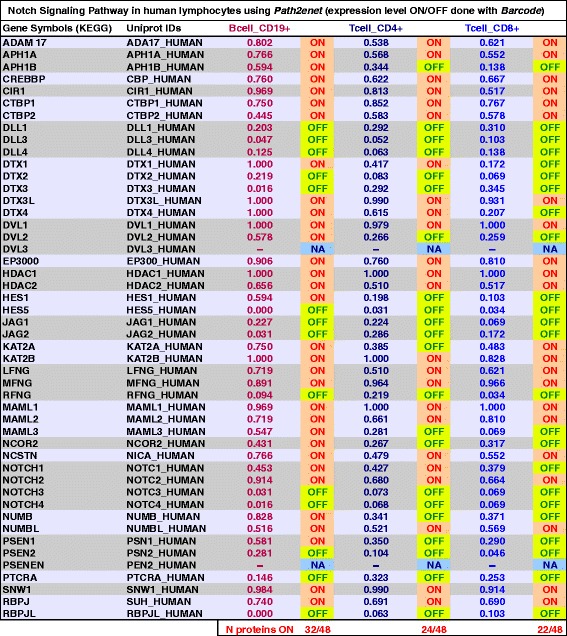
Results of the analysis performed for the NOTCH Signaling Pathway (including 48 genes) with *Path2enet* based on the gene expression *Barcode* algorithm for the data sets of B cell CD19+, T cell CD4+ and T cell CD8+. The data sets correspond to 163 samples of expression microarrays. The treshold to indicate if a gene was expressed (ON) or (OFF) is 0.4. Genes labeled with NA are not present in the expression platform and thus could not be annotated

First, we needed to apply the gene expression *Barcode* analysis to the gene products present in the NOTCH pathway-network (Fig. 1b) to identify which nodes were active in these cell types. The quantitative results of these analyses are presented in Fig. 3. Using the treshold of 0.4 for the normalized expression, the B cell network expressed 34 of 48 of the NOTCH pathway proteins. In contrast, the T cell network expressed 22–24 of the NOTCH proteins. It was very interesting to show that in all lymphocytes DLL1/2/3 and JAG1/2 were absent (i.e., they were OFF). In fact, these proteins are ligands of the NOTCH receptors of lymphocytes coming from the external cells that connect to them, therefore they should not be present in the lymphocytes. This is clearly shown in the quantitative analysis (Fig. 3), since all these genes were labeled OFF (not expressed) in B cells and in T cells.

We also observed that the only NOTCH paralogs detected in the lymphocytes were NOTCH2 and some NOTCH1. It is well known that NOTCH2 is preferentially expressed in mature naive B cells and interacts with DTX1, thus playing an important role in B cell development [17]. We also saw that the level of DTX1 in B cells was much higher (DTX1 = 1.00) than in T cells CD4+ (0.41) or CD8+ (0.17) (Fig. 3). This result is also in agreement with several studies that have shown that T cells are normally developed in absence of DTX1 [18].

Finally, another differential protein found expressed in B cells but not in T cells was the transcription factor HES1. The presence and role of this transcription factor in lymphocytes has been proven in several studies [19, 20]. In fact, it has been indicated that in T cells HES1 is dispensable beyond the beta selection checkpoint [21]. This explains our detection of HES1 in B cells CD19+ and its abscence in T cells CD4+ and CD8 + .

As a whole the data presented in Figs. 2 and 3 were very consistent with our current knowledge of the role of the NOTCH pathway in human B and T lymphocytes, enhancing the value of generating well defined “pathway-expression-networks” for specific cell types which is the scope of *Path2enet*.

### *Path2enet* tool for pathways: usability and formats

KEGG pathways database (http://www.kegg.jp/) provides KGML files for each biological pathway on its website. For example, in the case of the human NOTCH signaling pathway (KEGG ID reference: hsa04330) the KGML file can be downloaded freely as “hsa04330.xml”. The link for this file is: http://www.kegg.jp/kegg-bin/download?entry=hsa04330&format=kgml. In this way, any specific pathway is accessible via its KGML file in the KEGG website and *Path2enet* R package provides functions to download these files and create a MySQL database derived from the KGMLs (as explained in the R vignette included with *Path2enet*). Moreover, to facilitate the use of the pathway KMGL files within the application *Path2enet*, we also provided an SQL dump file (“Path2enet_KeggSQL.sql”) generated with all the KMGL files of *Homo sapiens* (this datafile is provided at: http://bioinfow.dep.usal.es/path2enet/). This allows the creation of the necessary SQL database within the user’s computer to query for specific pathways and to use the other functions of *Path2enet*. This database resource is not just a compendium of KMGL files from KEGG given that it provides some important added values: (i) it includes a mapping of all the gene and protein identifiers (IDs) from KEGG to the IDs of *UniProtKB* (used as the reference protein database in *Path2enet*); (ii) it includes a relational SQL structure, based on the extracted data from the pathways, that allocates such information in two principal indexed tables: one describing the pair-wise links or relations between protein pairs, and another one describing the characteristics of each singular protein.

With respect to the use of other formats, other than XML and KGML, *Path2enet* can also use any database or resource provided in a “network structure” as an *igraph* object, because the tool includes functions to read and load in R *igraph* objects. For the use of other standard formats, such as SBML or BioPAX, there are already tools that address this scope. For example *KEGGtranslator* [22], an easy-to-use stand-alone application that can visualize and convert KGML formatted XML-files into multiple output formats. This tool supports a plethora of output formats, being able to increase the information in translated documents beyond the scope of the KGML document. *KEGGtranslator* converts KEGG files (KGML formatted XML-files) to SBML, BioPAX, SIF, SBGN, SBML-qual, GML, GraphML and LaTeX. Moreover, in *Bioconductor* (https://www.bioconductor.org/) there are packages to parse, modify and visualize BioPAX data, like *rBiopaxParser* [23] or *PaxtoolsR* [24]. At the moment, we are working on a workflow to use these packages to create SQL databases, similiar to the SQL described above, but using data from other pathway resources such as Reactome or Pathway Commons. This work is under development, but one of main problems in the use of these resources is not the use of standard formats, like BioPAX or SBML, but the accurate mapping to standard protein identifiers from UniProtKB.

## Conclusions

*Path2enet* produces pathway-expression-networks reading and integrating high quality pathway data, protein interaction data and expression cell specific data. The development of this type of tools can be very useful to achieve a more integrative and global view of the links and association between the proteins working in specific cellular systems. The tool is not restricted to predefined pathways since it can create large networks blending multiple layers of biological information. Moreover, the tool can use either pre-processed expression data from selected repositories or experimental expression data from RNA-Seq or microarrays.

In this study we applied *Path2enet* to the analysis of the NOTCH signaling pathway in B cells and T cells. We showed that the expression networks based on a large microarray data set of these samples are different for each cell type, modulating the original general view of the canonical pathway provided by KEGG. Moreover, the observed differences have clear biological meaning, as demonstrated, for example, when only 2 out of the 4 NOTCH paralog proteins (NOTCH1, 2, 3, 4) were expressed in B cells and T cells. Thus, a clear signal in all lymphocytes was observed for NOTCH2; while NOTCH1 was also detected in B cells CD19+ and in T cells CD4+. We also found that key regulators like DTX1 and HES1 are strongly expressed in B cells and less expressed, or not present, in T cells. All these results give support to the the value of the networks that *Path2enet* generates that are cell-type and context specific. In conclusion, users have the possibility to combine several pathways and include protein-protein interaction data to find key players in a specific biological context either for normal or for pathological samples.

## Additional files

Additional file 1: List of 163 high-density oligonucleotides expression microarrays from human B-cells and T-cells used in this study (taken from Gene Expression Omnibus, GEO, database). (DOCX 125 kb)
Additional file 2: R vignette provided as a guided tutorial to facilitate the installation and use of the *Path2enet* package. (HTML 9198 kb)
