# Supplementary material for: Path2enet: generation of human pathway-derived networks in an expression specific context
Source: BMC Genomics. 2016 Oct 25;17(Suppl 8):731. doi: 10.1186/s12864-016-3066-7 (PMC5088520; doi:10.1186/s12864-016-3066-7)
Supplement: Additional file 1: — List of 163 high-density oligonucleotides expression microarrays from human B-cells and T-cells used in this study (taken from Gene Expression Omnibus, GEO, database). (DOCX 125 kb) [file 12864_2016_3066_MOESM1_ESM.docx]

**Additional File 1**

List of 163 high-density oligonucleotides expression microarrays from human B-cells and T-cells used in this study (taken from Gene Expression Omnibus, GEO, database):

**B cells** (CD19+) 32 microarrays, samples ID in GEO (http://www.ncbi.nlm.nih.gov/geo/):

GSM595853 GSM595858 GSM595863 GSM595870 GSM609252 GSM609254 GSM609257 GSM609258 GSM609259 GSM609260 GSM609262 GSM609263 GSM609264 GSM609265 GSM609266 GSM609267 GSM629980 GSM629981 GSM705297 GSM705298 GSM705299 GSM705300 GSM705301 GSM746743 GSM746744 GSM746745 GSM746746 GSM746747 GSM746748 GSM746750 GSM746751 GSM746752

**T cells** (CD4+) 96 microarrays, samples ID in GEO (http://www.ncbi.nlm.nih.gov/geo/):

GSM251101 GSM251105 GSM251110 GSM251111 GSM251114 GSM251126 GSM251129 GSM251192 GSM251194 GSM304415 GSM304420 GSM304945 GSM304946 GSM322864 GSM345116 GSM345117 GSM345118 GSM345119 GSM345120 GSM345121 GSM345122 GSM345123 GSM345124 GSM345125 GSM345126 GSM345127 GSM345128 GSM345129 GSM345130 GSM345131 GSM345132 GSM345133 GSM345134 GSM345135 GSM345136 GSM345137 GSM345138 GSM345139 GSM345140 GSM345141 GSM345142 GSM345143 GSM345144 GSM345145 GSM345282 GSM345283 GSM345284 GSM364915 GSM364916 GSM371639 GSM371640 GSM371641 GSM371646 GSM371652 GSM372721 GSM372722 GSM372723 GSM372724 GSM372725 GSM372726 GSM372727 GSM372728 GSM372729 GSM372730 GSM403596 GSM413790 GSM413792 GSM413795 GSM413796 GSM413798 GSM413800 GSM413803 GSM413804 GSM472023 GSM548000 GSM548001 GSM595854 GSM595859 GSM595864 GSM595871 GSM642302 GSM642303 GSM642304 GSM705302 GSM705303 GSM705304 GSM705305 GSM705306 GSM788303 GSM788304 GSM788305 GSM788306 GSM788307 GSM788308 GSM788309 GSM788310

**T cells** (CD8+) 35 microarrays, samples ID in GEO (http://www.ncbi.nlm.nih.gov/geo/):

GSM159405 GSM159406 GSM198958 GSM371708 GSM371709 GSM371710 GSM371711 GSM371712 GSM372731 GSM372732 GSM372733 GSM372734 GSM372735 GSM372736 GSM372737 GSM372738 GSM372739 GSM372740 GSM372741 GSM403597 GSM595856 GSM595861 GSM595865 GSM595872 GSM705312 GSM705313 GSM705314 GSM705315 GSM705316 GSM826755 GSM826757 GSM826758 GSM826760 GSM826761 GSM826763
