# Supplementary material for: Path2enet: generation of human pathway-derived networks in an expression specific context
Source: BMC Genomics. 2016 Oct 25;17(Suppl 8):731. doi: 10.1186/s12864-016-3066-7 (PMC5088520; doi:10.1186/s12864-016-3066-7)
Supplement: Additional file 2: — R vignette provided as a guided tutorial to facilitate the installation and use of the Path2enet package. (HTML 9198 kb) [file 12864_2016_3066_MOESM2_ESM.html]

Path2enet


# Path2enet

# **Generation of human pathway-derived networks in an expression specific context.**

- 1. Introduction
  - Translating biological pathways into protein networks
  - Pathways and protein-protein interactions
  - Gene expression data
    - Pre-processed data
    - Experimental datasets
- 2. Installation
  - Setting up ID mapping tables
  - Setting up MySQL
  - Setting up KEGG PATHWAY database
  - Setting up PPI database
  - Setting up EST UniGene database
- 3. Using R to access and search into the databases
  - Access the Pathways database
  - Access the PPI database
- 4. Setting up the expression resources
  - Generate tissue specific datasets based on UniGene EST database
  - Setting up gene expression Barcode dataset
  - Setting up RNA-Seq dataset
- 5. Generating pathway-expression-networks
  - Normal and dynamic representation of the graphs
  - Tissue specific representation of the graphs
- 6. Integration of experimental expression data
- 7. Acknowledgments
- 8. References

```
## Warning: package 'knitr' was built under R version 3.1.3
```

AUTHORS: **Conrad Droste** and **Javier De Las Rivas**

Bioinformatics and Functional Genomics Group

Cancer Research Center (CiC-IBMCC, CSIC/USAL/IBSAL)

37007 Salamanca, SPAIN.

CONTACT: *jrivas(AT)usal(.)es* - AND - *conrad.droste(AT)usal(.)es*

# 1. Introduction

Large-scale and small-scale experiments regarding the interaction of genes as DNA and RNA units, proteins and other molecular components within the cells are producing large amounts of **interaction data**, which are stored in many different biomolecular databases. The human interactome, for example, is composed of around 20,000 protein-coding genes, around 1,000 metabolites and a still undefined number of distinct proteins and functional RNA molecules (Vidal, Cusick & Barabasi, 2011). In total, this sums up to more than 100,000 cellular components expected to form the biomolecular **interactome** in human cells. These components are related to each other in different ways. The number of relations and functional associations exceeds substantially the number of components, making the interactome a complex system difficult to depict and analyze. Despite this complexity, the nature of the cellular interactomes allows to render or transcribe them into **biomolecular networks**, that can integrate different layers of information to generate comprehensive relational spaces and provide better representation and view of the cellular systems. Moreover, the **networks** can be analyzed with computers to explore and quantify the centrality and the weight of the different partners, as well as to find clusters or modules of highly related elements. This is the main objective of developing the bioinformatic application tool here presented, called **Path2enet**.

**Path2enet** is a tool to generate biomolecular networks derived from biological pathways, integrated with protein-protein interaction data (PPI) and expression data in a cell specific context (i.e. using data from specific tissues, cell-types or experimental conditions).

**Path2enet** is developed as a **R package**, open source and open access, to allows the construction of **pathway-expression-networks** reading and integrating the information from biological pathways, protein interaction data and expression cell specific data.

The user can easily search for interactions in the pathways translated in networks by **Path2enet**. It is also possible to combine various pathways into *meta-pathways* presented as unified networks, providing a global view of the biological interactions that can occurr in a cell. Such global views avoid the separation between pathways that many times is done following arbitrary criteria.

After generating these specific **pathway-expression-networks**, the tool provides several visualization and analyzing features. In fact, **Path2enet** uses the R package *igraph* (Gabor *et al.*, 2006) to generate networks that can be loaded into *Cytoscape* software (Saito *et al.*, 2012). In conclusion, a clear goal of the tool is to help biological researchers to create individual networks which fullfill their specific cellular conditions and interpret their studies in a network based context.

## Translating biological pathways into protein networks

We have developed a bioinformatic tool called **Path2enet** that provides a translation of biological pathways in protein networks integrating several layers of information about the biomolecular nodes in a multiplex view. **Path2enet** is a R package that reads the relations and links between proteins stored in a comprehensive database of biological pathways, (using KEGG - Enciclopedia de Kyoto de Genes), and integrates it with expression data from various resources and with data about protein-protein physical interactions (using APID - Agile Protein Interactomes DataServer). **Path2enet** tool uses the expression data to decide if a given protein in a network (i.e. a node) is active (ON) or not (OFF) in a specific cellular context or sample type. In this way, **path2enet** reduces the complexity of the networks and reveals the proteins that are active (expressed) in specific conditions.

As a proof of concept, this work presents a practical “case of use” generating the **pathway-expression-networks** corresponding to the *Notch Signaling Pathway* in human B- and T-lymphocytes. This case is produced by the analysis and integration in **path2enet** of a experimental dataset of genome-wide expression microarrays produced with these cell types (i.e. B cells and T cells):

## Pathways and protein-protein interactions

**Path2enet** uses the following databases to obtain and build the main protein networks:

**KEGG PATHWAY**: KEGG PATHWAY is a curated pathway database which represents a high quality resource for biomolecular interactions and associations between proteins in a well-defined and known biological context. **Path2enet** uses the KGML-files of the KEGG database to upload and transform the pathway information into an interaction network (Kanehisa, 2000). To achieve this, the package creates a MySQL-database with all the information from known biological pathways, focusing on the protein pairs that are included in each pathway and the way that they are known to interact. In this way, **Path2enet** also uses the R-package *RMySQL* (James *et al.*, 2012) that allows connecting and handling the MySQL databases using R programming.

**APID** (Agile Protein Interactomes DataServer): APID is an interactive web server that provides unified generation and delivery of protein interactomes mapped to their respective proteomes. This resource is a new, fully redesigned server that includes a comprehensive collection of protein interactomes for more than 400 organisms (25 of which include more than 500 interactions) produced by the integration of only experimentally validated protein-protein physical interactions (Alonso-López *et al.*, 2016). APID is the second generation of a previous resource that integrated six primary databases of molecular interactions: BIND, BioGRID, DIP, HPRD, IntAct and MINT (Prieto & De Las Rivas, 2006). In this way, APID platform has been fully redesigned and updated this year, and **Path2enet** includes a human protein interactome from APID with 214,844 unique protein-protein interactions.

To achieve a correct unification of the different databases and resources described, the identifiers (IDs) taken and implemented in **Path2enet** as principal keys to integrate the data are the **UniProtKB** IDs (Magrane *et al.*, 2011) for proteins and for the corresponding protein-coding gene products. These are, for example for HRAS human protein: **UniProt ID** = HRAS\_HUMAN and **UniProt AC** = P01112. In this way, all the KEGG gene identifiers and ENSEMBL gene identifiers in the studied datasets are annotated to the **UniProt IDs** following the mapping tables provided by UniProt.

## Gene expression data

**Path2enet** uses several expression data and procedures to **estimate if a gene is present** (i.e. if it is active or not, ON / OFF) in a given specific tissue o cell type, in a given experiment:

### Pre-processed data

1. **UniGene ESTs (Expression Sequencing Tags)**. *Path2enet* uses the information of the UniGene database to include tissue specific gene expression data derived from ESTs (expressed sequence tags). This database, which is annotated to adult and normal state in *Homo sapiens*, includes **18,880 gene/protein** entries detected in **51 human tissues**.
2. **Gene expression Barcode**. *Path2enet* includes a large genome-wide expression resource derived from the analysis of hundreds of high-density oligonucleotide microarrays done using the algorithm *Barcode*. This resource stores information about the expression of **17,268 gene/protein** entries detected in **195 tissues and cell lines**. The dataset can be found at Barcode website (McCall *et al.*, 2011; McCall *et al.*, 2014).
3. **Human Body Map**. *Path2enet* also includes RNA-Seq data of the Human Body Map 2.0 that stores FPKM expression data of **18,744 gene/protein** entries in **16 human tissues**. These FPKMs fragments per kilobase of exon per million reads are calculated using *Cufflings* 2.2.0 algorithm (Trapnell *et al.*, 2012) and annotated to Ensembl GRCh37 with the R-package *Biomart* (Durnick *et al.*, 2009).
4. **Human Protein Atlas**. Finally, *Path2enet* includes another large RNA-Seq dataset produced by the Human Protein Atlas, which stores the FPKM expression data of **19,078 gene/protein** entries of **33 human tissues** (Uhlen *et al.* 2015).

### Experimental datasets

Beside the pre-processed expression datasets provided in several of the integrated resources, **Path2enet** uses the Gene Expression *Barcode* algorithm with the R package *fRMA* (McCall *et al.*, 2011; McCall *et al.*, 2014) to evaluate if a gene is **expressed** (i.e. **ON**, “active”, “present”) or **not expressed** (i.e. **OFF**, “not active” and therefore “not present”) in a set of samples. The users can also incorporate their own expression data and define the ON/OFF thresholds.

At present the *Barcode* algorithm works mainly with microarray data. However, filters to select only the expressed genes can be applied also using EST data (from UniGene) or RNA-Seq data (setting up an adequate threshold value that according to recent studies can be FPKM >= 2) (Roson-Burgo *et al.* 2015).

# 2. Installation

To install **Path2enet** type in your R console:

```
install.packages("Path2enet")
```

(NOTE: **Path2enet** has been tested to work with the following **R versions**: 3.1, 3.2, 3.3)

To make sure that all **Path2enet** functionalities are available, you will need to install the following packages (in case they are not automatically installed as dependencies):

```
source("http://bioconductor.org/biocLite.R")
biocLite(c("abind", "affy", "AnnotationDbi", "Biobase", "biomaRt", "DBI", "frma", "GenomeInfoDb", "ggm", "ggplot2", "graphics", "hgu133afrmavecs", "hgu133plus2frmavecs", "igraph", "ipred", "org.Hs.eg.db", "plotrix", "RCurl", "RMySQL", "reshape", "sna", "XML"))
```

## Setting up ID mapping tables

**Path2enet** package generates an **IDMappingTable** to map ENTREZ Gene IDs (identifiers) or KEGG IDs to UniProt IDs (i.e. **UniProt Entry** and **UniProt Entry Name**, which for example for **KRAS** are: **“P01116”** and **“RASK\_HUMAN”**, respectively). To do this, **Path2enet** uses the table that UniProt database provides here: ftp://ftp.uniprot.org/pub/databases/uniprot/current\_release/knowledgebase/idmapping/. This step is necessary to generate the databases and to integrate the different data sources with common primary IDs. In this way, the final and unique key identifiers of the generated networks are the **UniProt IDs**, to allow a correct integration and comparison of different datasets.

In the example, the function **IDmappingFunction** downloads the **IDMappingTable** for *Homo sapiens*. This function automatically downloads the necessary file and stores it in a *tmp* directory.

```
tmp <- tempdir()
referenceTable <- IDmappingFunction(directory=tmp, org="HUMAN")
```

In this way, the **IDMappingTable** for human is included as “referenceTable”.

```
data(referenceTable)
```

## Setting up MySQL

**Path2enet** package builds and uses a **MySQL relational database** to store and later access the data and information needed from the pathways. Therefore, it is necesarry to setup in your computer a **MySQL database** or to have access to an already installed SQL database. Further information and instructions about how to install and configure a MySQL database can be found at https://www.mysql.com/.

## Setting up KEGG PATHWAY database

These steps take some time depending on your equipment. But normally these steps have to be done only once at the beginning or when you want to update the pathways data.

The following function, named **KeggXML2SqlDatabase**, downloads the **KGML files** of the KEGG PATHWAY database and creates the MySQL database that we call: “KeggSQL”.

```
data(referenceTable)
KeggXML2SqlDatabase(user="USER", host="IP/localhost", driver="MySQL", password="PW", directory="/path/to/store/KGMLfiles/", database="KeggSQL",  tricode="hsa", getKGML=TRUE, referenceTable=referenceTable)
```

If you have downloaded the **KGML files** previously at folder “Keggbrfile”, you can use these files to create the MySQL database that we call: “KeggSQL”.

```
data(referenceTable)
KeggXML2SqlDatabase(user="USER", host="IP/localhost", driver="MySQL", password="PW", directory="/path/to/KGMLfiles/", database="KeggSQL",  tricode="hsa", kegbr="/path/to/Keggbrfile/", referenceTable=referenceTable)
```

As another way to facilitate the instalation and use of the pathways in MySQL, we also provide a **SQL dump file** (called “**Path2enet\_KeggSQL.sql**”) that includes all the biological pathways for human (*Homo sapiens*) and was created with Path2enet package using the corresponding KMGL files. In this way, you can install the SQL database using the MySQL software of your computer (remember that it is necesarry to have a **MySQL database**).

The **SQL dump file** named “**Path2net\_KeggSQL.sql**” is located in http://bioinfow.dep.usal.es/path2enet/

Once it is installed it creates the database called: “**KeggSQL**”.

The way to install the **SQL dump file** in a computer (working in the command-line terminal) is the following:

```
[person@computer home]$ mysql -u user -p 
mysql> drop database if exists KeggSQL;
mysql> create database KeggSQL;
mysql> use KeggSQL;
mysql> source Path2net_KeggSQL.sql;

OR

[person@computer home]$ mysql -u user -p 
mysql> drop database if exists KeggSQL;
mysql> create database KeggSQL;
mysql> exit;
[person@computer home]$ mysql -u user -p KeggSQL < Path2net_KeggSQL.sql
```

This database resource is not just KEGG, since it includes important added values: **(i)** it includes a mapping of all the gene and protein identifiers that KEGG provides to the IDs of UniProtKB, that is taken as the reference protein database in **Path2enet**; **(ii)** it includes a relational SQL structure that extracted the information from the pathways to place it in two principal indexed tables, one describing the pair-wise links or relations between protein pairs and another one describing the characteristics of each sigular protein.

## Setting up PPI database

The protein-protein interaction network from APID is stored in **data(ppiHumanUniP)**. In this step we load this dataset and create a protein-protein interaction database in MySQL.

```
data(ppiHumanUniP)
Apid2Sql(user="USER", host="IP/localhost", driver="MySQL",password="PW", ppi=ppiHumanUniP, database="PPI")
```

## Setting up EST UniGene database

The UniGene database stores information on Expression Sequencence Tags (ESTs). The EST data give an overview about which genes are expressed in specific human tissues and cell types, because if one specific gene does not have any EST in one specific tissue it indicates that it is not present in such tissue.

The implemented function **mkESTdb** downloads and generates the information in a new database in MySQL. The parameters to create the database are the same as for the **KeggXML2SqlDatabase** function. The name of the species has to fit the name the UniGene database and *species* input has to be the abbrevation that the UniGene database uses. Look at ftp://ftp.ncbi.nih.gov/repository/UniGene/ to find the correct name and abbrevation.

This R code generates an EST MySQL database for *Homo sapiens*:

```
mkESTdb(dbDriver = "MySQL", dbUser = "root", dbHost = "localhost", password = NULL, directory = tempdir(), dbData = "Unigene", ESTdataexits = FALSE, name = "Homo_sapiens", species = "Hs.")
```

# 3. Using R to access and search into the databases

**Path2enet** tool allows access and searching in the SQL databases generated. Here, we explain:  
 **(i)** How to find relations or interactions in the pathway database?  
 **(ii)** How to find interactions in the protein-protein-interaction database?

## Access the Pathways database

The user can search with KEGG identifiers [KeggIDs], pathway identifiers (like for example “hsa04330”), pathway titles or UniProt identifiers [UniprotIDs].

```
kegglist <- c('hsa:11317' , 'hsa:5986' , 'hsa:51107' , 'hsa:55851' , 'hsa:5663' , 'hsa:5664' , 'hsa:9541' , 'hsa:55534' , 'hsa:84441' , 'hsa:9794' , 'hsa:1487' , 'hsa:1488', 'hsa:1488', 'hsa:3065', 'hsa:3066' )

searchFunction(dbDriver="MySQL", dbUser="USERNAME", dbHost="IP/localhost", dbName="KeggSQL", password="PW", dbData="Path2net_KeggDatabase", KeggID=kegglist, selectALL='TRUE',  Local="FALSE")
```

Searching for relations or links between the genes in the “signal transduction pathways” from KEGG:

```
searchFunction(dbDriver="MySQL", dbUser="USERNAME", dbHost="IP/localhost", dbName="KeggSQL", password="PW", dbData="Path2net_SignalPathwayrelation", KeggID=kegglist, selectALL='TRUE',  Local="FALSE", Relations="TRUE")
```

Searching with a list of proteins using UniProt IDs:

```
genelist <- c( "4EBP1_HUMAN", "AAPK1_HUMAN" ,"AAPK2_HUMAN" ,"AKT1_HUMAN" , "AKT2_HUMAN" , "AKT3_HUMAN" , "AKTS1_HUMAN" ,"I4E1B_HUMAN" ,"IF4E2_HUMAN" ,"IF4E_HUMAN" , "MTOR_HUMAN",  "TSC2_HUMAN" , "P55G_HUMAN" ,"P85A_HUMAN")

signal.compl <- searchFunction(dbDriver="MySQL",  dbUser="USERNAME", dbHost="IP/localhost", dbName="KeggSQL", password="PW", dbData="Path2net_SignalPathwayrelation", print="FALSE" , GeneID=genelist , Relations="TRUE")

#Select only interactions

signal.geneID <- searchFunction(dbDriver="MySQL",  dbUser="USERNAME", dbHost="IP/localhost", dbName="KeggSQL", password="PW", dbData="Path2net_SignalPathwayrelation", print="FALSE" , GeneID=genelist , Relations="TRUE", selectGeneID="TRUE")
```

## Access the PPI database

It is possible to search for interactions in the PPI database (obtained from APID).

```
genelist <- c( "4EBP1_HUMAN", "AAPK1_HUMAN" ,"AAPK2_HUMAN" ,"AKT1_HUMAN" , "AKT2_HUMAN" , "AKT3_HUMAN" , "AKTS1_HUMAN" ,"I4E1B_HUMAN" ,"IF4E2_HUMAN" ,"IF4E_HUMAN" , "MTOR_HUMAN",  "TSC2_HUMAN" , "P55G_HUMAN" ,"P85A_HUMAN")

#Select only interactions

signal.geneID <- searchFunction(dbDriver="MySQL", dbUser="root", dbHost="IP/localhost", dbName="PPI", password="PW", dbData="PROTEINPAIR", GeneID=genelist, selectALL='TRUE',  Local="FALSE", Relations="TRUE", selectGeneID="TRUE")
```

# 4. Setting up the expression resources

This section shows how to load or create tissue specific datasets to generate tissue specific networks.

## Generate tissue specific datasets based on UniGene EST database

In the step above, we created the EST database “UniGene”.  
 In the next step we can access this database and create a new EST dataset which fits our analisis. In the example we only select ESTs which correspond to tissues annotated to “adult” stage and that correspond to “normal” data (i.e. not to disease related states):

```
data(referenceTable)
estTissueUniP <- createEstByTissue(dbDriver="MySQL", dbHost="localhost", dbUser="root", password=NULL, dbData="Unigene", cancer_source="normal", developmental_stage="adult", referenceTable=referenceTable)
estTissueUniP <- unigene$est_by_uniprotidtissue
```

By setting the “check”" parameter to TRUE, we can do a query to see which “developmental stages” and which “normal/disease states” are in UniGene:

```
stages <- createEstByTissue(dbDriver="MySQL", dbHost="localhost", dbUser="root", password=NULL, dbData="Unigene", check="TRUE")
```

**Path2enet** package also includes an EST dataset previously built with the **createEstByTissue** function. In this vignette we will select and use ESTs annotated only to “normal” and “adult”:

```
data(estTissueUniP)
```

## Setting up gene expression Barcode dataset

The data from Barcode that **Path2enet** incorporates is based on the “HGU133plus2 (Human) tissues v3” file of The Gene Expression Barcode 3.0 (“abc-tis-gpl570-formatted\_v3.csv”). The genes are mapped to proteins with the function **referenceTableHGU133Plus** from the package. To do this, first, this function uses a custom CDF (“chip definition file”) from BRAINARRAY tool that maps the probesets from the *Affymetrix* HGU133 plus 2.0 array to Ensemble Gene IDs (CDF file named “HGU133Plus2.HS.ENSG”). Second, it uses the **IDMapping** table from UniProt to map from gene IDs to UniProt IDs. Further information is included in the help function.

```
data(barcodeTissueUniP)
help(barcodeTissueUniP)
```

## Setting up RNA-Seq dataset

As it is mentioned above, **Path2enet** uses two RNA-Seq datasets: from Human Body Map 2.0 and from Human Protein Atlas. Both are produced with Illumina sequencing platforms.

- for **Human Body Map 2.0**: The tool takes the ftp://ftp.ensembl.org/pub/release-70/bam/homo\_sapiens/genebuild/ \*.bam archives, and uses cufflinks (on human genome GRCh37) to generate the FPKM values of the genes based on the ENSEMBL gene IDs. These gene IDs are then mapped to UniProt protein IDs with fucntion **referenceTable**.
- for **Human Protein Atlas**: The tool takes the “rna\_tisse.csv.zip” file (available at http://www.proteinatlas.org/about/download website). Again, the gene IDs are then mapped to UniProt protein IDs with fucntion **referenceTable**.

Further information is included in help function.

```
data(rnaseqTissueUniP)
help(rnaseqTissueUniP)
```

# 5. Generating pathway-expression-networks

The main function of the package, called **path2enet**, is the one that creates the tissue specific networks. In the example reported the function generates a network for the human **Notch Signaling Pathway** (this is the “local network”). Furthermore, the function can look for any other interaction of the proteins from the Notch pathway in all the other KEGG pathways (only in the pathways annotated to “normal”, i.e. not annotated to any disease) to generate what we called the “global network”. At the same time, it can incorporate to the network the protein-protein interactions from the PPI database included.

```
graphs <- path2enet(dbDriver="MySQL", dbUser="root", dbHost="IP/localhost", password="PW", dbName="KeggSQL", Pathwaytitle="Notch Signaling Pathway", Local="FALSE", dbData="Path2net_SignalPathwayrelation", dbGlobal="Path2net_Normal",  dbPPI="PPI", unitissue=Unitissue, barcode=barcodeTissueUni,  rnaseq=rnaseqTissueUniP)
```

The output named “graphs” (produced after running **path2enet**) includes two lists: the list “Graphs”, which includes the generated networks, and “Analysed\_Graphs”, which includes the calculated parameters of the networks.

```
names(graphs)
[1] "Graphs"          "Analysed_Graphs"
names(graphs$Graphs)
[1] "PPI_PATHWglobal"        "PATHWlocal_PATHWglobal" "PPI_PATHWlocal" 
names(graphs$Analysed_Graphs)
[1] "EdgeList_PATHWlocal_withGroups"                  "EdgeList_withSubtype_withoutGroups"             
[3] "Analysed_PATHWlocal_KeggIDs_withGroups"          "Analysed_PATHWlocal_UniprotKBIds_withGroups"    
[5] "Analysed_PATHWlocal_UniprotKBIds_withoutGroups"  "Analysed_PPI"                                   
[7] "Analysed_PATHWglobal_UniprotKBIds_withGroups"    "Analysed_PATHWglobal_UniprotKBIds_withoutGroups"
```

The R documentation about **path2net** function explains all the different objects and tables that it generates.

```
help(path2enet)
```

In the selected example, we continue with the **Notch Signaling Pathway** and the network based on its nodes in the PPI database.

```
graph.NotchPPi <- graphs$Graphs$PPI_PATHWlocal$PATHWlocal2PPI
```

## Normal and dynamic representation of the graphs

The **graphTKplotterPATHW** function uses the graph object that we named “graph.NotchPPi”" and draws the network. The bold edges in the network are relations from the original **Notch Signaling Pathway** in KEGG. The thin edges represent new relations between nodes derived from the PPI database which were not annotated in KEGG.

```
graph.NotchPPi <- graphs$Graphs$PPI_PATHWlocal$PATHWlocal2PPI

#Normal plot
graph.NotchPPi.plot <- graphTKplotterPATHW(graph.NotchPPi)
#Dynamic plot
graph.NotchPPi.plot <- graphTKplotterPATHW(graph.NotchPPi, tkplot=TRUE)
#Save Layout: Select the ID of the tkplot device
layout_graphtk <-tkplot.getcoords(tkp.id=ID)
#Use the Layout
graph.NotchPPi.plot <- graphTKplotterPATHW(graph.NotchPPi, tkplot=TRUE, layout=layout_graphtk)
```

## Tissue specific representation of the graphs

Using the gene expression data described above either from ESTs, Barcode and microarrays or RNA-Seq, **Path2enet** generates tissue or cell type specific networks. To do this, the user can select various **vertex.attributes** and set a filter value based on the type of expression attribute (“est”, “barcode”, “rnaseq”“): **(i)** If a gene shows an expression value higher than the selected treshold, it is considered as ON-active. **(ii)** If the value is lower, it is considered as OFF-inactive.

```
NotchPPi.attributes <- list.vertex.attributes(graph.NotchPPi)
NotchPPi.attributes[grep("liver",NotchPPi.attributes)]
# "est_liver"       "barcode_liver"   "rnaseq_liver_bm" "rnaseq_liver_pa"

graphs.est.liver <- graphTKplotterTissue(graph.NotchPPi,value=0 ,tissue="est_liver", name = "EST liver", sub = "Subtitle ESTs liver", tkplot = "TRUE", pdf = "FALSE", directory = NULL)

graphs.barcode.liver <- graphTKplotterTissue(graph.NotchPPi,value=0 ,tissue="barcode_liver", name = "Barcode liver", sub = "Subtitle Barcode liver", tkplot = "TRUE", pdf = "FALSE", directory = NULL)
```

It is also possible to filter for various expression types at once and combine the results for a expression network of higher confidence. For each kind of a dataset, the user can select a specific threshold. The function generates a graph object only containing nodes, which have expression values above the selected threshold.

```
liver.attributes <- NotchPPi.attributes[grep("liver",NotchPPi.attributes)]
NotchPPi.liver <- graphParameters(graph.NotchPPi, vertex.attributes=liver.attributes, barcode.value=0,est.value=0,rnaseq.value=0)
NotchPPi.liver.graph <- NotchPPi.liver$Reducedgraph

graphs.est.liver <- plot(NotchPPi.liver.graph, edge.color=E(NotchPPi.liver.graph)$color)
```

All selected proteins had an expression level in the samples studied higher value than the tresholds:

# 6. Integration of experimental expression data

Besides the pre-processed datasets, **Path2enet** enables to integrate experimental expression data as well. In the case study here presented, we use the algorithm to generate specific **pathway-expression-networks** of the **Notch signaling pathway** in three types of human cells: B cells (CD19+) and T cells (CD4+ and CD8+).

In this example we download 4 microarray data for each cell type and perform a comparison of the results for the B cells (CD19+) versus the T cells (CD4+ and CD8+). First, we download the .CEL files and create the **AffyBatch** R objects:

```
# Normal human T cells CD4+ from GSE12079
cd4 <- c("GSM304415", "GSM304420", "GSM304945", "GSM304946")
dir.create(path="./TCELLCD4/")
for(cd4_file in cd4){
  getGEOSuppFiles(GEO=cd4_file,makeDirectory=FALSE, baseDir="./TCELLCD4/")
}
files.dir <- dir("./TCELLCD4/")
lapply(paste("./TCELLCD4/",files.dir[grep("CEL.gz", files.dir)], sep=""), function(x) gunzip(x))
tcell_cd4 <- ReadAffy(celfile.path="./TCELLCD4/")
pData(tcell_cd4)[,1] <- "TCELLCD4+"

# Normal human T cells CD8+ from GSE14879
cd8 <- c("GSM371708", "GSM371709","GSM371710", "GSM371711")
dir.create(path="./TCELLCD8/")
for(cd8_file in cd8){
  getGEOSuppFiles(GEO=cd8_file,makeDirectory=FALSE, baseDir="./TCELLCD8/")
}
files.dir <- dir("./TCELLCD8/")
lapply(paste("./TCELLCD8/",files.dir[grep("CEL.gz", files.dir)], sep=""), function(x) gunzip(x))
tcell_cd8 <- ReadAffy(celfile.path="./TCELLCD8/")
pData(tcell_cd8)[,1] <- "TCELLCD8+"

tcell_cd4_cd8 <- merge.AffyBatch(tcell_cd4,tcell_cd8)

# Normal human B cells from GSE24223
bcell_cd19 <- c("GSM595853", "GSM595858", "GSM595863", "GSM595870")
dir.create(path="./BCELLCD19/")
for(bcd19 in bcell_cd19){
  getGEOSuppFiles(GEO=bcd19,makeDirectory=FALSE, baseDir="./BCELLCD19/")
}
files.dir <- dir("./BCELLCD19/")
lapply(paste("./BCELLCD19/",files.dir[grep("CEL.gz", files.dir)], sep=""), function(x) gunzip(x))
bcell_cd19 <- ReadAffy(celfile.path="./BCELLCD19/")
pData(bcell_cd19)[,1] <- "BCELLCD19+"

bcell_cd19_tcell_cd4_cd8 <- merge.AffyBatch(bcell_cd19, tcell_cd4_cd8)
```

In the next step, we use the **expr2barcode** function to perform an analisis with the Barcode algorithm and integrate the data into the **Notch Signaling Pathway** that was generated above, object “graph.NotchPPi”. We map the *Affymetrix* probesets to Uniprot IDs using **referenceTableHGU133Plus**. For further information about this mapping: “help(referenceTableHGU133Plus)”.

```
data(referenceTableHGU133Plus)

expr2barcode.result.bcell_cd19_tcell_cd4_cd8 <- expr2barcode(exprAffy=bcell_cd19_tcell_cd4_cd8, phenotype="sample",referenceTable=referenceTableHGU133Plus,  igraph=graph.NotchPPi, cutoff=1)
```

The generated object *expr2barcode.result.bcell\_cd19\_tcell\_cd4\_cd8* includes four lists:  
 - *BarcodeGraph* includes the igraph object with the integrated results of the analisis.  
 - *BarcodeIndividual* includes the results of the barcode analisis of each microarray.  
 - *BarcodePheno* the mean of the barcode values of the phenotypes.  
 - *AffyBatchUniprot* expression set mapped to Uniprot-IDs.

```
bcell_cd19_tcell_cd4_cd8_graph <- expr2barcode.result.bcell_cd19_tcell_cd4_cd8$BarcodeGraph

bcell_cd19_barcode_graph  <- graphTKplotterTissue(bcell_cd19_tcell_cd4_cd8_graph, tissue="barcode_Pheno_BCELLCD19+", tkplot=TRUE, value=0.4)

#Shows the On/Off state based on the selected treshold. 
head(bcell_cd19_barcode_graph$state_OnOff)

tcell_cd4_barcode_graph  <- graphTKplotterTissue(bcell_cd19_tcell_cd4_cd8_graph, tissue="barcode_Pheno_TCELLCD4+", tkplot=TRUE, value=0.4)

tcell_cd8_barcode_graph  <- graphTKplotterTissue(bcell_cd19_tcell_cd4_cd8_graph, tissue="barcode_Pheno_TCELLCD8+", tkplot=TRUE, value=0.4)
```

However, **Path2enet** package also includes a graph with the complete dataset corresponding to the analisis of the B cells (CD19+) and the T cells (CD4+ and CD8+), stored in the data object: “path2enet.BCELLCD19\_TCELLCD8\_TCELLCD4\_NOTCHLOCAL\_expr2barcode”.

```
data(path2enet.BCELLCD19_TCELLCD8_TCELLCD4_NOTCHLOCAL_expr2barcode)

bcell_cd19_tcell_cd4_cd8_graph <- path2enet.BCELLCD19_TCELLCD8_TCELLCD4_NOTCHLOCAL_expr2barcode$BarcodeGraph

bcell_cd19_barcode_graph  <- graphTKplotterTissue(bcell_cd19_tcell_cd4_cd8_graph, tissue="barcode_Pheno_BCELL_CD19+", tkplot=TRUE, value=0.4)

#Shows the On/Off state based on the selected treshold. 
head(bcell_cd19_barcode_graph$state_OnOff)

tcell_cd4_barcode_graph  <- graphTKplotterTissue(bcell_cd19_tcell_cd4_cd8_graph, tissue="barcode_Pheno_TCELLCD4+", tkplot=TRUE, value=0.4)

tcell_cd8_barcode_graph  <- graphTKplotterTissue(bcell_cd19_tcell_cd4_cd8_graph, tissue="barcode_Pheno_TCELLCD8+", tkplot=TRUE, value=0.4)
```

# 7. Acknowledgments

**Bioinformatics and Functional Genomics Group**.  
 Cancer Research Center (CiC-IBMCC, CSIC/USAL/IBSAL).  
 Consejo Superior de Investigaciones Cientificas (CSIC).  
 Universidad de Salamanca (USAL).  
 Salamanca, Spain.

We acknowledge the funding and grants provided to Dr. J. De Las Rivas group by the Local Government, “Junta de Castilla y Leon” (JCyL, Valladolid, Spain, grant number BIO/SA08/14) and the Spanish Government, “Ministerio de Economia y Competitividad” (MINECO, ISCiii, Madrid, Spain, grants number PI12/00624 and PI15/00328). We also acknowledge a PhD research grant to Conrad Droste (“Ayudas a la contratacion de Personal Investigador”) provided by the JCyL with the support of the “Fondo Social Europeo” (FSE).

# 8. References

- Alonso-López D, Gutiérrez MA, Lopes KP, Prieto C, Santamaría R, De Las Rivas J: **APID interactomes: providing proteome-based interactomes with controlled quality for multiple species and derived networks.** *Nucleic Acids Res.* 2016, pii:**gkw363**.
- Csardi G, Nepusz T: **The igraph software package for complex network research.** *InterJournal Complex Systems.* 2006, **1695**:1-9.
- De Leeuw WC, Rauwerda H, Jonker MJ, Breit TM: **Salvaging Affymetrix probes after probe-level re-annotation.** *BMC Res Notes.* 2008, **1**:66.
- Durinck S, Spellman PT, Birnez E, Huber W: **Mapping identifiers for the integration of genomic datasets with the R/Bioconductor package biomaRt.** *Nat Protoc.* 2009, **4**:1184-91.
- James DA, Debroy S: **RMySQL: R interface to the MySQL database.** R package version 0.9-3. 2012. http://CRAN.R-project.org/package=RMySQL
- Kanehisa M, Goto S: **KEGG: kyoto encyclopedia of genes and genomes.** *Nucleic Acids Res.* 2000, **28**:27-30
- Magrane M, UniProt Consortium: **UniProt Knowledgebase: a hub of integrated protein data.** *Database.* 2011, **2011**:bar009.
- McCall MN, Uppal K, Jaffee HA, Ziliox MJ, Irizzarry RA: **The Gene Expression Barcode: leveraging public data repositories to begin cataloging the human and murine transcriptomes.** *Nucleic Acids Res.* 2011, **39**:D1011-5
- McCall MN, Jaffee HA, Zelisko SJ, Sinha N, Hooiveld G, Irizzarry RA, Ziliox MJ: **The Gene Expression Barcode 3.0: improved data processing and mining tools.** *Nucleic Acids Res.* 2014, **42**:D938-D943.
- Prieto C, De Las Rivas J: **APID: Agile Protein Interaction DataAnalyzer.** *Nucleic Acids Res.* 2006, **34**:W298W302.
- Roson-Burgo B, Sanchez-Guijo F, Del Cañizo C, De Las Rivas J: **Transcriptomic portrait of human Mesenchymal Stromal/Stem Cells isolated from bone marrow and placenta.** *BMC Genomics.* 2014, **15**:910.
- Saito R, Smoot ME, Ono K, Ruscheinski J, Wang PL, Lotia S, Pico AR, Bader GD, Ideker T: **A travel guide to Cytoscape plugins.** *Nature Methods.* 2012, **9**:1069-1076.
- Trapnell C, Hendrickson DG, Sauvageau M, Goff L, Rinn JL, Pachter L: **Differential analysis of gene regulation at transcript resolution with RNA-Seq.** *Nat Biotechnol.* 2012, **31**:46-53.
- Uhlen M, Fagerberg L, Hallstrom BM, Lindskog C, Oksvold P, Mardinoglu A, Sivertsson A, Kampf C, Sjostedt E, Asplund A, Olsson I, Edlund K, Lundberg E, Navani S, Szigyarto CA, Odeberg J, Djureinovic D, Takanen JO, Hober S, Alm T, Edqvist PH, Berling H, Tegel H, Mulder J, Rockberg J, Nilsson P, Schwenk JM, Hamsten M, von Feilitzen K, Forsberg M, Persson L, Johansson F, Zwahlen M, von Heijne G, Nielsen J, Ponten F: **Tissue-based map of the human proteome.** *Science.* 2015, **347**:1260419.
- Vidal M, Cusick ME, Barabasi AL: **Interactome networks and human disease.** *Cell.* 2011, **144**:986-98.
